# Short and long-term effects of pulmonary rehabilitation in interstitial lung diseases: a randomised controlled trial

**DOI:** 10.1186/s12931-018-0884-y

**Published:** 2018-09-20

**Authors:** Silvia Perez-Bogerd, Wim Wuyts, Veronica Barbier, Heleen Demeyer, Alain Van Muylem, Wim Janssens, Thierry Troosters

**Affiliations:** 1 Erasme University Hospital, Chest Department, Université Libre Bruxelles, B-1060 Brussels, Belgium; 2 University Hospitals Leuven, Department of Respiratory Diseases, B-3000 Leuven, Belgium; 3 KU Leuven, Department of Rehabilitation Sciences, B-3000 Leuven, Belgium; 4 KU Leuven, Department of Chronic Diseases, Metabolism and Ageing, B-3000 Leuven, Belgium

## Abstract

**Background:**

Few data are available on the long-term effect of pulmonary rehabilitation (PR) and on long PR programs in interstitial lung diseases (ILD).

We aimed to evaluate the effects of PR on exercise capacity (6-Minute Walking Distance, 6MWD; Peak Work Rate, W_max_), quality of life (St George’s Respiratory Questionnaire, SGRQ), quadriceps force (QF) and objectively measured physical activity in ILD after the 6-month PR-program and after 1 year.

**Methods:**

60 patients (64 ± 11 years; 62% males; 23% with IPF) were randomly assigned to receive a 6 month-PR program or usual medical care.

**Results:**

Exercise capacity, quality of life and muscle force increased significantly after the program as compared to control (mean,95%CI[ll to ul]; 6MWD + 72,[36 to 108] m; W_max_ 19, [8 to 29]%pred; SGRQ − 12,[− 19 to − 6] points; QF 10, [1 to 18] %pred). The gain was sustained after 1 year (6MWD 73,[28 to 118] m; Wmax 23, [10 to 35]%pred; SGRQ − 11,[− 18 to − 4] points; QF 9.5, [1 to 18] %pred). Physical activity did not change.

**Conclusions:**

PR improves exercise tolerance, health status and muscle force in ILD. The benefits are maintained at 1-year follow-up. The intervention did not change physical activity.

**Trial registration:**

Clinicaltrials.gov NCT00882817.

**Electronic supplementary material:**

The online version of this article (10.1186/s12931-018-0884-y) contains supplementary material, which is available to authorized users.

## Background

Interstitial lung diseases (ILD) are a group of disorders characterized by progressive dyspnoea, exercise limitation and poor quality of life [1, 2]. Treatment typically involves corticosteroids or cytotoxic drugs and, in some cases, antifibrotic agents [3]. While these therapies tackle the respiratory problems to some degree, strategies that further reduce complaints, improve health status and delay extra-pulmonary disease progression are therefore needed.

It has been shown that quadriceps weakness [4] and physical inactivity [5] are present in idiopathic pulmonary fibrosis (IPF) and poor exercise capacity, physical inactivity and, recently, a low fat-free mass index have been related to mortality [5–7]. Therefore, enhancing exercise tolerance, physical activity and muscle function in IPF must be treatment goals that were not shown to be altered by pharmacotherapy [3]. These factors have been proven to be tackled by pulmonary rehabilitation (PR) in other chronic lung diseases, such as COPD [8].

Rare randomised controlled trials exist about the effectiveness of PR in ILD [9–13]. A Cochrane review [14] accumulated 168 patients for the most studied outcome measure (6-min walking distance, 6MWD) and reported clinically significant benefits on this outcome after short programs (8–12 weeks). A recent large Australian trial reported somewhat smaller effects [9]. None of these studies propose long programs and only one small trial showed limited long-term effects (beyond 6 months) [15].

The aim of this study was to evaluate the feasibility and effectiveness of a longer (6-month) multidisciplinary PR program on exercise tolerance, muscle strength, quality of life and physical activity in a randomized controlled trial. In addition, long-term benefits of the program were investigated.

## Methods

### Study subjects

Patients with chronic ILD referred to the ILD clinic in the University Hospital of Leuven were considered for enrolment. According to eligibility criteria, potential candidates were informed about the protocol and written informed consent was obtained, between March 2009 and September 2011. The study was approved by the local ethics committee (B32220095560) of this hospital.

Inclusion criteria were a diagnosis of ILD according to internationally established criteria with a formal workup and multidisciplinary discussion [1, 2], dyspnoea on exertion and having a stable medical therapy with no infection/exacerbation in the previous 4 weeks.

Exclusion criteria were comorbidities (unstable angina, recent myocardial infarction or cerebrovascular accident, active cancer, severe orthopedic disorders) or systemic manifestations (active myopathy, arthralgia, synovitis) that do not allow training and a life expectancy below 3 months.

### Study design

Patients were randomly assigned to the rehabilitation or control group using sealed envelopes prepared and shuffled before the start of the study by an independent person unrelated to the study protocol. The envelope was opened by the allocator sequentially, only after the participant’s name was written on it.

Patients assigned to the rehabilitation group were invited to attend a 6-month outpatient rehabilitation program with a total of 60 sessions, 3 times per week for the first 3 months and thereafter twice weekly. The program was performed in accordance with the guidelines [16] and is detailed in the Additional file 1 (online data supplement). Patients assigned to the control group were treated with maximal medical care and with an identical medical follow-up, similarly to the active intervention arm.

### Assessments

All measurements were made at enrolment and at 3, 6 and 12 months after the start of the study.

The primary outcome of the study, functional exercise capacity, was measured as the distance walked during the best of two 6-minute walking tests (6MWD) [17] and also expressed as percentage of the predicted values [18]. Use of oxygen was standardized to a minimal flow rate of 2 l-per-minute and subsequent follow-up tests were conducted in the same conditions as at baseline. The difference in 6MWD between treatment and control group at 6 months was considered as the primary endpoint.

The secondary outcomes were forced vital capacity (FVC); slow vital capacity (SVC); diffusing capacity for carbon monoxide (DL_CO_); arterial partial pressure of oxygen (PaO_2_); maximal work rate (W_max_); 6MWD; quadriceps (QF) and hand grip (HF) muscle force; quality of life evaluated by the the St George’s respiratory (SGRQ) and the chronic respiratory disease questionnaires (CRQ) and dyspnoea (MRC 1 to 5). More details on the assessments are provided in the Additional file 1 (online data supplement).

Patients were provided with an activity monitor SenseWear Armband (Bodymedia, Pittsburgh, USA), in order to assess objectively their physical activity. They were instructed to wear the monitor for at least 7 consecutive days, from waking up until going to bed. Days with less than 8 h of wearing time during waking (defined as 7 AM-8 PM) were excluded. Only week days were used to minimize the variability. A valid measurement was defined as having at least 2 valid weekdays. The influence of the duration of the daylight time was included as a covariate [19].

Mean step count and mean time in at least moderate intense physical activity (MPA) were chosen as the physical activity outcomes. MPA was defined as any activity with an energy expenditure above 3 METs [20]. The SenseWear has been validated in COPD patients [21], a respiratory population with a comparable inactive lifestyle.

Physical activity at 1 year was not included in the analysis due to the large number of missing data at this time point, caused by incompliance of patients and technical problems. For this reason the data were not judged as being representative of the entire sample and therefore not included in the analyses.

All adverse events during the rehabilitation program were registered.

### Statistics

All variables were expressed as means ± standard deviation (SD) or 95% confidence interval (95% CI). A minimal 6MWD benefit of 38 ± 43 m might be expected after 3 months [10]. To anticipate a similar difference of 40 ± 45 m, a sample size of 20 patients in each arm is needed to show a statistically significant difference at the 0.05 p-level with 80% power. Anticipating on 40% of dropouts, a total number of 60 patients were randomized. Statistical analysis was performed using mixed models including the outcomes as measured in the 4 visits (baseline, after 3, 6 and 12 months). ‘Visit’ was included as a class variable. *P* values below 0.05 were considered as significant in all analyses (R version 3.2.1) [22]. Group, time and group*time interactions were retrieved. This interaction term assesses the pure effect of rehabilitation since it is the difference, at each time-point, between changes from baseline in the rehabilitation group and in the control group. Duration of daylight was including as a (time varying) covariate when investigating the physical activity outcomes [19]. The number needed to treat (NNT) to achieve an increase in functional exercise capacity was defined as a clinically significant improvement (> 30 m) in the 6MWD.

## Results

### Study recruitment and population characteristics

**Fig. 1:**
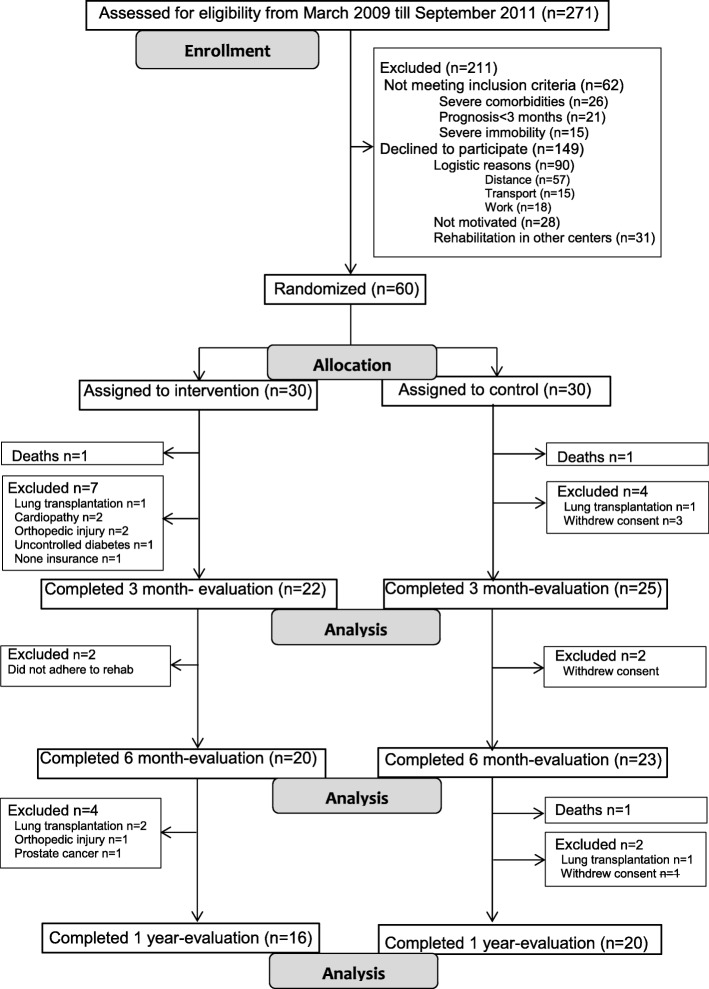
Consort Flow Diagram

Figure 1 shows the Consort flow diagram. From 271 patients referred to the ILD clinic, 209 were eligible for inclusion; 60 (22%; 37 M/23F) were randomized.

Sixty-seven percent of patients completed the rehabilitation program and 60% of the randomized patients were followed up at 1 year.

Table 1 shows the baseline characteristics of the participants. At baseline, 6MWD (expressed as percentage of the predicted value) was slightly lower (*p* = 0.045) and hypersensitivity pneumonitis was more represented in the rehabilitation group. The groups were matched for other parameters (Table 1, Additional file 1: Table S1). Patients who dropped out did not differ from those who completed the study in the rehabilitation group; in the control group, drop-outs appeared to be younger and to have worse diffusing capacity (Additional file 1: Table S2).

**Table 1:** Baseline characteristics

		*Control* *n* = 30	*Rehabilitation* *n* = 30	*p- Value*
Age (years)		64 (8)	64 (13)	0.81
Gender (men)		15 (50%)	22 (73%)	0.06
BMI (Kg/m^2^)		26 (5)	28 (4)	0.21
Diagnosis				
IIP		10 (33%)	11 (37%)	0.79
• IPF		7 (23%)	7 (23%)	1.0
• NSIP		3 (10%)	3 (10%)	1.0
• DIP		0	1 (3%)	0.50
KNOWN CAUSES		12 (40%)	16 (53%)	0.30
• Chronic HP		4 (13%)	12 (40%)	**0.02**
• Asbestosis		0	1 (3%)	0.50
• Drug induced ILD		1 (3%)	1 (3%)	1.0
• CTD-ILD		7 (23%)	2 (7%)	0.14
UNCLASSIFIABLE ILD		8 (27%)	3 (10%)	0.09
Steroids		14 (47%)	15 (50%)	0.80
SVC	(L)	2.6 (1.1)	2.9 (0.7)	0.35
	(%pred)	77 (22)	76 (20)	0.86
DL_CO_	(mmol.min^−1^.kPa^− 1^)	3,4 (1,1)	4 (1,6)	0.11
	(%pred)	41 (13)	45 (16)	0.30
PaO_2_ (mmHg)		77 (14)	76 (11)	0.71
6MWD	(m)	491 (95)	462 (123)	0.32
	(%pred)	79 (14)	71 (16)	**0.045**
W_max_ (%pred)		71 (26)	61 (20)	0.10
QF (%pred)		81 (36)	78 (23)	0.70
HF (%pred)		85 (27)	89 (23)	0.61
SGRQ total (points)		40 (18)	42 (14)	0.68
Physical Activity (PA)				
• Steps per day		7182 (3523)	5745 (3312)	0.12
• MPA		57 (21–158)	36 (10–130)	0.14

Data expressed as mean (SD) or numbers and MPA as geometric mean (geometric interval): geometric mean is antilog(m) and geometric interval is (antilog(m-SD) – antilog(m + SD)), m and SD being the mean and the standard deviation of the log-transformed MPA, respectively. *BMI* body mass index, *IIP* idiopathic interstitial pneumonia, *IPF* idiopathic pulmonary fibrosis, *NSIP* nonspecific interstitial pneumonia, *DIP* desquamative interstitial pneumonia, *HP* hypersensitivity pneumonitis, *CTD-ILD* connective tissue disease-related ILD, *SVC* slow vital capacity, *DLco* diffusion capacity for carbon monoxide, *PaO*_*2*_ partial pressure of oxygen at breathing room air, *6MWD* six-minute walking distance, *W*_*max*_ maximal workload, *QF* quadriceps force, *SGRQ* St George's respiratory questionnaire, *MPA* moderate intense physical activity (dailt time spent in activities with an intensity of at least 3 METs)a. *p*-values in bold indicate a statistically significant difference between intervention and control group

### Effects

**Fig. 2:**
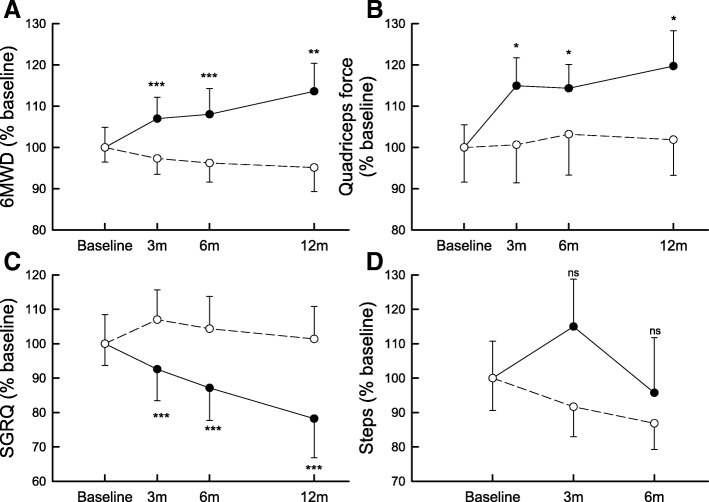
Effects of PR and at 1-year on exercise capacity, muscle force, health status, physical activity. 6-min walking distance (6MWD) (Panel **a**), quadriceps force (Panel **b**), St-Georges respiratory questionnaire (SGRQ) (Panel **c**) and steps per day (Steps) (Panel **d**) expressed as percentage (%) of the baseline value as a function of time. Closed circles (solid lines) and open circles (dashed lines) are the mean values and SEM at each time points of rehabilitation and control groups, respectively. Rehabilitation and control groups were compared for rehabilitation effects at each time points: *** *p* < 0.001; ** *p* < 0.01; * *p* ≤ 0.05; ns *p* > 0.05

At 6 months, the 6MWD of the intervention group was mean, 95% CI [ll to ul] + 72, [36 to 108]m better as compared to the usual care group (Table 2, Fig. 2). The benefit was largely maintained between groups at 1-year follow-up (73, [28 to 118] m). Taking a conservative intention to treat approach where none of the dropouts in either group is counted as a responder, 11 patients (37% of the initially selected 30 patients) had a clinically significant improvement in their 6MWD at the 1-year time point, whereas only 5 (17%) of the controls had such an improvement. This results in a NNT of 5.

**Table 2:** Effects of PR and at 1-year on exercise capacity, muscle force and health status

	*Control*	*Rehabilitation*	*Rehabilitation* *effect mean (95% CI ll,ul)*	*p- Value*
6MWD (m)				
Baseline	491 (57)	462 (57)		0.36
3 months	474 (57)	504 (57)	59 (33, 85)	**< 0.001**
6 months	468 (57)	511 (57)	72 (36, 108)	**< 0.001**
1 year	456 (57)	501 (57)	73 (28, 118)	**0.002**
W_max_ (%pred)				
Baseline	71 (18)	61 (18)		0.15
3 months	68 (18)	68 (18)	10 (2, 19)	**0.01**
6 months	62 (18)	71 (18)	19 (8, 29)	**< 0.001**
1 year	61 (18)	74 (18)	23 (10, 35)	**< 0.001**
SVC (%pred)				
Baseline	77 (21)	76 (21)		0.86
3 months	77 (21)	78 (21)	2 (−2, 6)	0.23
6 months	75 (21)	77 (21)	3 (−1, 7)	0.20
1 year	76 (21)	79 (21)	4 (−0.2, 9)	0.06
DL_CO_ (%pred)				
Baseline	42 (13)	45 (13)		0.31
3 months	39 (13)	44 (13)	1.2 (− 2.4, 4.8)	0.52
6 months	39 (13)	43(13)	0.9 (−3.6, 5.3)	0.70
1 year	38 (13)	45 (13)	3.2 (−1.8, 8.2)	0.21
QF (%pred)				
Baseline	81 (27)	78 (27)		0.69
3 months	81 (27)	88 (27)	10 (2, 18)	**0.01**
6 months	83 (27)	90 (27)	10 (1, 18)	**0.02**
1 year	88 (27)	94 (27)	9.5 (1, 18)	**0.04**
HF (%pred)				
Baseline	73 (17)	76(17)		0.56
3 months	76 (17)	91 (17)	12.5 (5, 20)	**0.002**
6 months	75 (17)	90 (17)	12 (4, 20)	**0.004**
1 year	79 (17)	88 (17)	6 (−3, 15)	0.20
SGRQ total				
Baseline	40 (14)	42 (14)		0.68
3 months	44 (14)	39 (14)	−7 (−13, −2)	**0.005**
6 months	46 (14)	35 (14)	−12 (−19, −6)	**< 0.001**
1 year	45 (14)	35 (14)	−11 (− 18, −4)	**0.002**

Data are expressed as mean (SD) from the mixed model. The rehabilitation effect at each time point is the difference (and its 95% confidence interval) between changes from baseline in the rehabilitation group and in the control group. *6MWD* six-minute walking distance, *W*_*max*_ maximal workload, *SVC* slow vital capacity, *DLco* diffusion capacity for carbon monoxide, *QF* quadriceps force, *HF* handgrip force, *SGRQ* St George’s respiratory questionnaire. The rehabilitation effect at each time point is the interaction term between time points and group effects. The *p*-Value on the baseline line assesses differences in baseline values, the other ones assess the effect of rehabilitation at each time point. *p*-values in bold indicate a statistically significance

Similarly, the intervention yielded significant increases in maximal exercise capacity (W_max_), health status (in all the domains) and quadriceps force at the end of PR (Table 2, Fig. 2, Additional file 1: Tables S3-S4), as compared to usual care. Most of these benefits were maintained at 1 year. A significant improvement was already shown after 3 months of PR for exercise tolerance, muscle force, total scores of health status, SGRQ activity domain and CRQ dyspnoea, emotion and mastery (Table 2, Additional file 1: Tables S3-S4).

The rehabilitation program did not change MRC dyspnoea and physical activity significantly as compared to the control group at any time point (Table 3, Fig. 2, Additional file 1: Table S4).

**Table 3:** Effects of PR on physical activity

	*Control*	*Rehabilitation*	*Rehabilitation* *effect mean (95% CI ll,ul)*	*p- Value*
Steps				
Baseline	7013 (2598)	5671 (2598)		0.10
3 months	6593 (2598)	5721 (2598)	470 (− 920, 1860)	0.50
6 months	6118 (2598)	5540 (2598)	764 (− 746, 2274)	0.32
MPA				
Baseline	54 (38, 78)	36 (25, 52)		0.46
3 months	44 (31, 64)	37 (26, 54)	1.3 (0.7, 2.2)	0.42
6 months	41 (29, 60)	33 (23, 48)	1.2 (0.6, 2.3)	0.57

Steps expressed as mean (SD) and MPA as geometric mean (geometric interval): geometric mean is antilog(m) and geometric interval is (antilog(m-SD), antilog(m + SD)), m and SD being the mean and the SD of the log-transformed MPA, respectively. *MPA* moderate intense physical activity (daily time spent in activities with an intensity of at least 3 METs). The rehabilitation effect at each time point is the difference (and its 95% confidence interval) between changes from baseline in the rehabilitation group and in the control group. As computation was made on log-transformed MPA, rehabilitation effect is expressed as a ratio (1.3 means + 30% in favor of rehabilitation). The *p*-Value on the baseline line assesses differences in baseline values, the other ones assess the effect of rehabilitation at each time point

PR did not influence lung function and arterial blood gases with the exception of forced vital capacity which was slightly better in the rehabilitation group as compared to usual care (Table 2, Additional file 1: Table S5).

#### Missing data

The mixed model assumes that a patient with a missing data follows the general trend of the group. We challenged this option by considering a more penalizing hypothesis: in the intervention group, missing data were assumed to follow the general trend of the control group whereas, in the control group, missing data were assumed to be equal to the baseline values. With this hypothesis, the PR-effect on 6MWD, W_max_, QF and CRQ remained significant at any time-point (Additional file 1: Table S6).

### Feasibility and adverse events

**Fig. 3:**
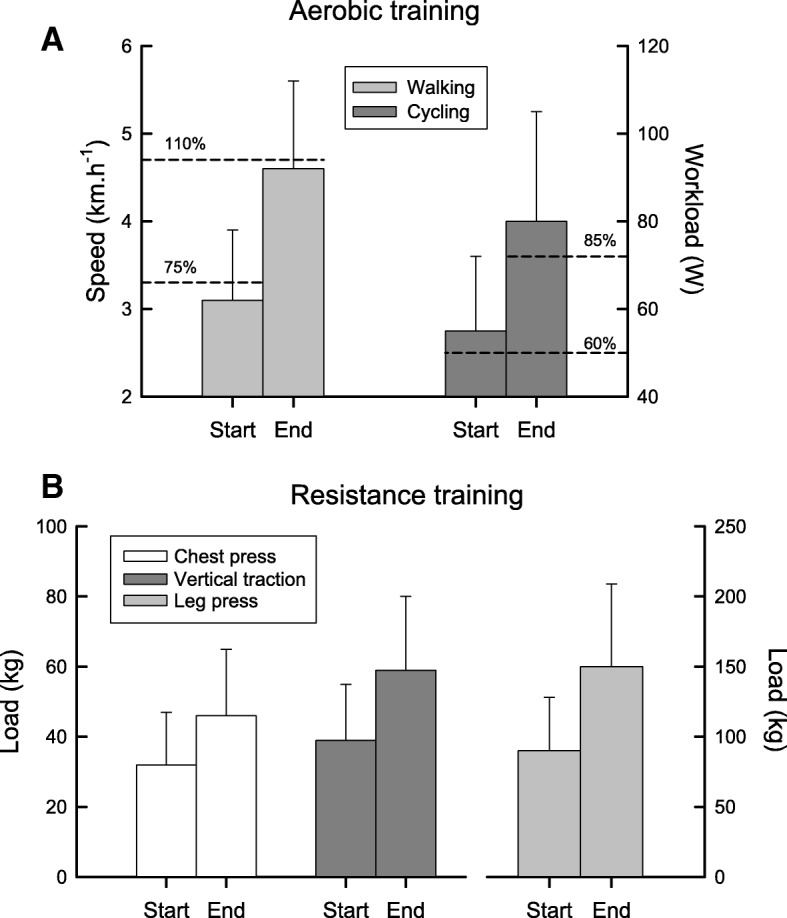
Feasability of the training. Training intensity at the Start and the End of the 6 month PR program, expressed as mean (SD). Aerobic training: patients started the Cycling at 60% of the initial maximal workload on the cycle ergometer and the Walking at 75% of their maximal walking speed during the initial 6-min walking test. Based on subjective Borg scale scores, the intensity was progressively increased up to 85% of the maximal workload and up to 110% of the maximal walking speed (Dashed lines, Panel **a**). Resistance training: patients started the program on a multi-gym device in 3 series of 8 repetitions at 70% of the initial 1-Repetition Maximum (1RM) load for each muscle group (chest press, vertical traction, leg press) and this load was progressively incremented (Panel **b**)

The patients participated in a mean of 50 ± 13 sessions. Aerobic training intensity for cycling started at 65 ± 8% of the initial W_max_. Walking was initiated at 72 ± 8% of the baseline mean 6MWD speed. These intensities were progressed to 95 ± 17% and 106 ± 15%, respectively. The resistance training load also increased significantly over time (Fig. 3).

No adverse events related to the exercise training were recorded during the PR program.

## Discussion

This is the first trial showing long-term benefits (at 1-year follow-up) after a 6-month outpatient PR program in ILD and the first study investigating the PR-effect on objectively measured physical activity (PA). As hypothesized, exercise capacity, health status and muscle force improved significantly after PR; these benefits were maintained after 1 year in those patients that completed the program. PR did not change the physical activity level.

The improvements in exercise capacity (6MWD) and quality of life (total scores; symptoms and impact of SGRQ; and dyspnea, emotion and mastery of CRQ) are relevant, as they exceeded the minimal clinically important difference (MCID) after PR and at 1-year follow-up (Table 2 and Additional file 1: Table S4) [23–26]. In IPF, the MCID of the 6MWD (22–45 m) [23, 26] and the SGRQ (5–8 points) [27] have been established [24], but, to our knowledge, the MCID of the CRQ has not been validated yet in ILD, only in COPD (0,5 points per item) [25]. Clinically relevant strategies, such as pulmonary rehabilitation, are very important for ILD patients whose quality of life is seriously deteriorated.

### Short-term benefits

To our knowledge, there are only 5 randomized controlled trials [9–13] and a meta-analysis [14] demonstrating improvements in exercise performance and symptoms after exercise training in ILD. Only one non-randomised study showed a benefit on muscle force after PR [28]. Overall, the magnitude of the effect in our trial was more pronounced than in previous shorter studies [10, 11, 13, 28–33].

Unfortunately, the large benefits after PR in ILD were not translated in objective gains in PA. This is in line with research in COPD [34]: some trials showed an increase in PA after PR and others failed to do so. In the negative studies, the lack of improvement was also observed despite gains in physiological function and health status. This is the first trial measuring the PR-effect on PA in ILD objectively. Previously, 2 studies [6, 35] have evaluated IPF patients with PA questionnaires and have suggested small benefits. However, questionnaires may not provide a reliable estimation of true PA behaviour. In our study, no specific interventions were introduced to coach patients towards more PA. Future studies could consider for instance the provision of feedback on PA using step-counters providing promising results in COPD [36].

### Feasibility

The high number of attended sessions and the high exercise intensity reached during the training sessions indicate that PR is feasible to be implemented in ILD in a similar way as in COPD [8]. In the present study, ILD patients trained alongside patients with COPD and other chronic respiratory conditions. However, it remains a point of attention that many patients refuse the offer for rehabilitation and 33% dropped out during the program although the reasons were not related to the provided intervention.

All ILD patients used oxygen supplements per protocol in order to avoid desaturation [37] and all the exercise tests were performed with oxygen in order to better show the improvements related to the training [38].

### Long-term benefits

The novel finding of this trial is the maintenance of the benefits in exercise capacity, health status and muscle force at 1 year in a subgroup of patients. So far, 5 studies [10, 13, 15, 28, 39] have evaluated long-term effects. Only 1 randomized trial [15] did find a discrete gain in quality of life at 1 year and one non-randomized study [39] showed an improvement on 6MWD and health status at 6 months.

The larger and long-term benefits in our study could potentially be explained by the differences in the rehabilitation program, the differences in disease aetiology and disease severity.

First, our regimen proposed a longer training than reported previously [9–13, 28–30, 32, 33, 35, 39]. In COPD, longer programs showed to have more effects than shorter programs [8, 40]. In addition, in line with recommendations for PR [16], a multidisciplinary approach consisting of occupational therapy, psychosocial and nutritional support was offered in our program.

Second, the lower proportion of IPF patients (23% in our trial compared to 100% [11–13] or around 50% of the patients [9, 10] in the other 5 randomised trials) could explain the more pronounced treatment effects. Indeed, the magnitude of improvement following PR in IPF was less than other ILD [10, 41, 42] or COPD [28]. However, a mixed population reflects more the clinical reality of most of ILD units.

Third, greater PR-benefits were described in patients with less functional impairment [9, 10, 28]. Our patients had better baseline 6MWD and muscle force [10, 11, 13, 28–33], whereas, the impairment in health status was comparable [43]. In fact, one trial with a similar baseline 6MWD did find similar PR-gains [12]. However, this remains controversial since other studies found greater improvements in the most disabled patients [29, 39].

Finally, it is important to note that, among the immunosuppressive medications proposed in the majority of non-IPF [2] and the recent recommended antifibrotic agents in IPF [3], none of them did increase exercise capacity, only pirfenidone has demonstrated to reduce the decline in 6MWD [44–47]; and nintedanib [48, 49] showed a discrete, not clinically significant, improvement in quality of life in 1 of the 2 phase three trials [49]. The fact that PR showed a large and maintained benefit on health status and exercise tolerance and without any clinically meaningful side effects is of utmost importance to ILD patients.

### Natural evolution of the disease

In contrast to previous trials, our study did not show a lung function decline over time in the whole group [9, 10]. This finding might be due to our larger amount of non-IPF patients.

Curiously, our study showed an improvement over time of muscle force in the entire group. Steroid therapy contributes to muscle dysfunction in ILD [50], a treatment often proposed to non-IPF patients [1, 2, 51]. In our study, 47% were under prednisolone at baseline or during the preceding 6 months (Table 2) and only 28% at 1 year. We suggest that muscle dysfunction of our ILD patients might be partly explained by the steroid therapy and the improvement over time by the gradual steroid tapering.

### Limitations

First, an a priori expected limitation of the study was the dropout rate (33%) over the course of the 6-month PR program. This number was slightly higher than observed in ILD following some shorter programs [10–13, 30, 33, 39], but similar as the Australian trial [9] and long programs in COPD [8, 40]. However, since the patients who dropped out from the rehabilitation group were very similar to those who completed the PR program, this has likely had minimal impact on the main results of the study. In addition, it is important to see that long-term benefits were obtained in a significant fraction of patients in the rehabilitation group resulting in a very acceptable NNT of 5 for the outcome 6MWD. Finally, a treatment of missing data penalizing the PR-effect did not affect the significance at any time-point.

Second, assessors were not blinded for 6MWD (main outcome) which may have led to an overestimation of the benefit, however this probably will not have affected the results of quality of life.

Third, only 20% from the eligible candidates were randomized in the present study (Fig. 1). This may compromise the external validity of the study. Whereas it shows the general difficulty of performing randomised controlled trials in rare diseases like ILD and patients may have considerable travel distances to attend our centre of reference. This has likely not impacted on the observed differences between groups.

## Conclusion

We conclude that a 6-month outpatient pulmonary rehabilitation program resulted in improvements in functional and maximal exercise capacity, health status and muscle force but not in physical activity in patients with ILD. The benefits were maintained at 1-year follow-up.

## Additional file

Additional file 1: Online data supplement contains **Table S1.** Baseline characteristics. **Table S2.** Baseline values of dropouts compared to non-dropouts patients at 1-year. **Table S3.** Effects of PR and at 1-year on maximal exercise capacity and muscle force. **Table S4.** Effects of PR and at 1-year on quality of life. **Table S5.** Effects of PR and at 1-year on lung function. **Table S6.** Effects of PR and at 1-year with penalizing treatment of missing data. (DOCX 57 kb)

## Abbreviations

1RM: One-Repetition maximum
6MWD: Six-minute walking distance
CRQ: Chronic respiratory disease questionnaire
CTD-ILD: Connective tissue disease-related ILD
DIP: Desquamative interstitial pneumonia
DL_CO_: Diffusion capacity for carbon monoxide
HF: Handgrip force
HP: Hypersensitivity pneumonitis
IIP: Idiopathic interstitial pneumonia
ILD: Interstitial lung diseases
IPF: Idiopathic pulmonary fibrosis
MPA: Moderate to intense physical activity
NNT: Number needed to treat
NSIP: Nonspecific interstitial pneumonia
PA: Physical activity
PaO_2_: Partial pressure of oxygen at breathing room air
PR: Pulmonary rehabilitation
QF: Quadriceps force
SGRQ: St-George’s respiratory questionnaire
VC: Slow vital capacity
W_max_: Maximal workload
