# Supplementary material for: Short and long-term effects of pulmonary rehabilitation in interstitial lung diseases: a randomised controlled trial
Source: Respir Res. 2018 Sep 20;19:182. doi: 10.1186/s12931-018-0884-y (PMC6149060; doi:10.1186/s12931-018-0884-y)
Supplement: Supplementary file 1 — Online data supplement contains Table S1. Baseline characteristics. Table S2. Baseline values of dropouts compared to non-dropouts patients at 1-year. Table S3. Effects of PR and at 1-year on maximal exercise capacity and muscle force. Table S4. Effects of PR and at 1-year on quality of life. Table S5. Effects of PR and at 1-year on lung function. Table S6. Effects of PR and at 1-year with penalizing treatment of missing data. (DOCX 57 kb) [file 12931_2018_884_MOESM1_ESM.docx]

Short and long-term effects of pulmonary rehabilitation in interstitial lung diseases: a randomised controlled trial

Silvia Perez-Bogerd^1,2^, MD, Wim Wuyts^2^, MD, PhD, Veronica Barbier^2^, MSc, Heleen Demeyer^3^, PhD, Alain Van Muylem^1^, PhD, Wim Janssens^2,3^, MD, PhD, Thierry Troosters^2,3^, PhD

**Pulmonary Rehabilitation program**

Our program consisted of 90 minutes of exercise training (cycling, treadmill walking, arm cranking, stair climbing and peripheral muscle training) and 30 minutes rotated by session of patient education, occupational therapy, nutrition counseling or psychosocial support**.** Patients started the cycling at 60% of the initial maximal work rate (W_max_) on the cycle ergometer and the walking at 75% of their maximal walking speed during the 6-minute walking test. Based on Borg scale scores, workload was progressively incremented up to 85% of the W_max_ and up to 110 % of the maximal walking speed. If needed interval exercise training was applied. Stair climbing was performed in 2-minute blocks (1 to 3 repetitions). Peripheral muscle training was performed on a multi-gym device in 3 series of 8 repetitions with the initial load set at 70% of the 1-Repetition-Maximum (1RM) for each muscle group (triceps, latissimus dorsi, pectoralis, quadriceps). Physiotherapists did ensure close supervision and continuous encouragement. Oxygen saturation, heart rate and dyspnoea Borg Score were measured during the training sessions and all patients trained with supplemental oxygen.

**Assessments**

**Pulmonary Function Test (PFT) and Arterial Blood Gases**

A spirometry (Sensor Medics, Anaheim, CA, USA), a body plethysmography (Body Box 1085, Medical Graphics, Inc., St. Paul, Minnesota) and a diffusing capacity for carbon monoxide (DL_CO_) measure were conducted according to the European Respiratory Society guidelines for pulmonary function testing [1-3]. The DL_CO_ was assessed by the single breath method (Sensor Medics 6200, Bilthoven, The Netherlands). Results were expressed as a percentage of the predicted normal values [4]. In addition, arterial blood gases were obtained by radial artery puncture and immediately analysed with the Synthesis 15 Blood gas analyser (Instrumentation Laboratory Lexington, MA, USA).

**Exercise Capacity**

Functional exercise capacity was measured by a 6-minute walking test (6MWT) [5] as the distance walked during the best of two 6-minute walking tests and was also expressed as percentage of the predicted normal values [6]. Maximal exercise capacity was assessed by an electrically braked cycle ergometer (Ergometrics 900, Ergoline, Bitz, Germany) with electrocardiographic monitoring under the supervision of a physician. This incremental cardiopulmonary exercise test was performed as previously described [7] and under supplemental oxygen, standardized at an inspiratory fraction of 30% O_2_ in all the patients. Maximal work rate (W_max_) was defined as the greatest work rate that the subject was able to maintain for more than 30 seconds and was compared with predicted values [8].

**Peripheral and Respiratory Muscle Force**

Isometric quadriceps strength was measured using a Cybex II dynamometer (Computer Sports Medicine Inc, Stoughton, MA, USA). Peak extension torque was evaluated at 60 degrees of knee flexion and the best value of three reproducible tests was related to reference values developed in our laboratory [9]. Isometric hand grip strength was assessed using a hand-held dynamometer (JAMAR, Preston, Jackson, Michigan, USA). The evaluation of peak force was made with the elbow flexed at 90° and fixed to the rib cage with the wrist in neutral position. The highest value of 3 reproducible measurements was expressed as a percentage of the predicted reference value [10]. Inspiratory and expiratory muscle strength was defined as the maximal mouth-pressure sustained for at least one second against a closed mouthpiece [11]. Maximal expiratory pressure (MEP) was evaluated from total lung capacity (TLC) and maximal inspiratory pressure (MIP) was assessed from residual volume (RV). The highest of at least five measurements was expressed as a percentage of the predicted values [12].

**Health-related Quality of Life**

The chronic respiratory disease questionnaire (CRQ) and the St George’s respiratory questionnaire (SGRQ) were used to assess health-related quality of life. Both of them were initially designed for COPD, they have been validated in ILD patients [13, 14] and are available in Dutch. The CRQ [15] includes 4 dimensions (Dyspnoea, Fatigue, Emotion and Control) and the SGRQ [16][17] consists of 3 domains (Symptoms, Activities, Impact). These questionnaires are not specifically developed for ILD. Recently a dedicated version of SGRQ for IPF has become available[18].

**Tables.**

**Table S1.** Baseline characteristics.

|  | *Control*  *(n=30)* | *Rehabilitation*  *(n=30)* | *p-Value* |
| --- | --- | --- | --- |
| Height (cm) | 166 (9) | 170 (9) | 0,08 |
| Weight (Kg) | 73 (15) | 81 (17) | 0,052 |
| Charlson index score | 1,3 (1,4) | 1,6 (1,3) | 0,45 |
| Charlson index score  (age-dependent) | 3,2 (1,6) | 3,5 (2,2) | 0,64 |
| FVC (L) | 2,6 (1,1) | 2,8 (0,7) | 0,42 |
| (%pred) | 79 (23) | 77 (21) | 0,78 |
| FEV_1_ (L) | 2 (0,6) | 2,2 (0,5) | 0,23 |
| (%pred) | 77 (17) | 76 (18) | 0,92 |
| FEV_1_/FVC | 80 (10) | 80 (8) | 0,85 |
| K_CO_ (mmol/min/KPa) | 1 (0,2) | 1,1 (0,4) | 0,32 |
| (%pred) | 72 (17) | 79 (28) | 0,27 |
| TLC (L) | 4 (1,5) | 4,3 (0,9) | 0,31 |
| (%pred) | 69 (18) | 69 (14) | 0,87 |

| W_max_  (W) | 85 (34) | 86 (17) | 0,92 |
| --- | --- | --- | --- |

| QF (Nm) | 106(39) | 128 (45) | 0,053 |
| --- | --- | --- | --- |
| HF (N) | 278 (117) | 316 (101) | 0,19 |
| MIP (cmH_2_O) | 83 (30) | 84 (30) | 0,82 |
| (%pred) | 93 (30) | 88 (24) | 0,53 |
| MEP (cmH_2_O) | 127 (44) | 151 (47) | **0,048** |
| (%pred) | 86 (24) | 95 (26) | 0,15 |
| CRQ (points) | 86 (22) | 88 (14) | 0,88 |
| Dyspnoea | 19(5) | 18(5) | 0,61 |
| Fatigue | 16(5) | 17(4) | 0,51 |
| Emotion | 31(9) | 30(8) | 0,64 |
| Mastery | 20(6) | 20(3) | 0,69 |
| SGRQ (points) | 40(18) | 42(14) | 0,68 |
| Symptoms | 43(24) | 52(24) | 0,17 |
| Activities | 54(21) | 55(21) | 0,84 |
| Impact | 33(20) | 32(13) | 0,84 |

Data expressed as mean (SD) and numbers. FVC: forced vital capacity, FEV_1_: forced expiratory volume in 1 second, K_CO_: transfer factor for carbon monoxide, TLC: total lung capacity, W_max_: maximal workload, QF: quadriceps force, HF: handgrip force, MIP: maximal inspiratory pressure, MEP: maximal expiratory pressure, CRQ: chronic respiratory disease questionnaire, SGRQ: St George’s respiratory questionnaire.

**Table S2.** Baseline values of dropouts compared to non-dropouts patients at 1-year.

|  | *Control* | | | | | *Rehabilitation* | | | | |
| --- | --- | --- | --- | --- | --- | --- | --- | --- | --- | --- |
|  | *Dropout*  n=10 | | *Non-dropout*  n=20 | | *p* | *Dropout*  n=14 | | *Non-dropout*  n=16 | | *p-Value* |
| Age (y) | 60 | (7) | 67 | (8) | **0,03** | 65 | (15) | 63 | (13) | 0,70 |
| Gender | 5F/ | 5M | 10F/ | 10M | 1 | 2F/ | 12M | 6F/ | 10M | 0,10 |
| Height (m) | 1,67 | (0,08) | 1,65 | (0,1) | 0,67 | 1,72 | (0,07) | 1,68 | (0,12) | 0,27 |
| Weight(Kg) | 68 | (16) | 76 | (14) | 0,18 | 80 | (15) | 83 | (18) | 0,62 |
| BMI(Kg/m^2^) | 24 | (5) | 28 | (4) | **0,05** | 27 | (4) | 29 | (5) | 0,14 |
| Diagnosis (IPF) | 1 | (10%) | 6 | (30%) | 0,37 | 4 | (29%) | 3 | (19%) | 0,67 |
| Steroids | 7 | (70%) | 8 | (40%) | 0,12 | 4 | (29%) | 10 | (62%) | 0,06 |
| FVC (L) | 2,56 | (1,66) | 2,59 | (0,76) | 0,96 | 2,7 | (0,82) | 2,84 | (0,54) | 0,56 |
| (%pred) | 72 | (34) | 82 | (15) | 0,37 | 71 | (22) | 83 | (18) | 0,11 |
| CV (L) | 2,58 | (1,65) | 2,67 | (0,72) | 0,87 | 2,77 | (0,81) | 2,93 | (0,58) | 0,54 |
| (%pred) | 70 | (32) | 81 | (15) | 0,30 | 70 | (20) | 82 | (18) | 0,08 |
| FEV_1_ (L) | 1,82 | (0,73) | 2,11 | (0,54) | 0,23 | 2,11 | (0,58) | 2,26 | (0,39) | 0,40 |
| (%pred) | 65 | (16) | 83 | (15) | **0,004** | 70 | (21) | 82 | (17) | 0,10 |
| FEV_1_/FVC | 79 | (15) | 81 | (7) | 0,80 | 79 | (8) | 80 | (8) | 0,77 |
| DL_CO_(mmol/min/KPa) | 2,85 | (1,07) | 3,65 | (1,18) | 0,08 | 3,93 | (2,12) | 4 | (1,12) | 0,91 |
| (%pred) | 33 | (10) | 46 | (12) | **0,01** | 43 | (18) | 47 | (13) | 0,46 |
| TLC (L) | 4,05 | (2,28) | 3,95 | (0,91) | 0,89 | 4,22 | (1,09) | 4,39 | (0,71) | 0,62 |
| (%pred) | 67 | (27) | 69 | (12) | 0,82 | 64 | (16) | 74 | (13) | 0,09 |
| 6MWD (m) | 485 | (86) | 494 | (101) | 0,80 | 458 | (149) | 466 | (100) | 0,86 |
| (%pred) | 73 | (11) | 82 | (15) | 0,13 | 68 | (20) | 73 | (12) | 0,42 |
| W_max_ (W) | 79 | (27) | 89 | (37) | 0,48 | 81 | (33) | 91 | (21) | 0,33 |
| (%pred) | 64 | (19) | 75 | (29) | 0,33 | 54 | (24) | 66 | (17) | 0,12 |
| QF (Nm) | 117 | (43) | 100 | (37) | 0,28 | 130 | (51) | 126 | (42) | 0,80 |
| (%pred) | 93 | (40) | 75 | (35) | 0,22 | 78 | (26) | 78 | (21) | 0,95 |
| HF (N) | 28,7 | (11,1) | 27,4 | (12,3) | 0,78 | 32,9 | (9,2) | 30,6 | (11) | 0,54 |
| (%pred) | 73 | (23) | 74 | (24) | 0,92 | 77 | (18) | 76 | (21) | 0,92 |
| CRQ total | 78 | (18) | 91 | (23) | 0,12 | 86 | (16) | 85 | (14) | 0,93 |
| Dyspnoea | 18 | (6) | 19 | (5) | 0,82 | 16 | (3) | 19 | (6) | 0,16 |
| Fatigue | 15 | (4) | 17 | (5) | 0,26 | 17 | (5) | 17 | (4) | 0,90 |
| Emotion | 24 | (7) | 34 | (8) | **0,007** | 33 | (8) | 29 | (8) | 0,21 |
| Control | 19 | (6) | 20 | (6) | 0,55 | 20 | (4) | 21 | (4) | 0,54 |
| SGRQ total | 44 | (26) | 36 | (16) | 0,33 | 42 | (15) | 42 | (13) | 0,89 |
| Symptoms | 54 | (23) | 39 | (21) | 0,08 | 49 | (26) | 54 | (23) | 0,62 |
| Activities | 57 | (28) | 53 | (17) | 0,63 | 55 | (20) | 56 | (22) | 0,87 |
| Impact | 44 | (22) | 27 | (16) | **0,02** | 34 | (14) | 30 | (12) | 0,44 |
| Steps | 6297 | (3958) | 6544 | (4230) | 0,89 | 4916 | (3542) | 6473 | (3445) | 0,28 |
| MPA | 50 | (11-229) | 54 | (23-124) | 0,90 | 41 | (11-146) | 42 | (12 -152) | 0,93 |

Data expressed as mean (SD) except for MPA represented as geometric mean (geometric interval): geometric mean is antilog(m) and geometric interval is (antilog(m-SD)- antilog(m+SD)), m and SD being the mean and the SD of the log-transformed MPA, respectively. BMI: body mass index, FVC: forced vital capacity, VC: slow vital capacity, FEV_1_: forced expiratory volume in 1 second, TLC: total lung capacity, DL_CO_: diffusion capacity for carbon monoxide, 6MWD: six-minute walking distance, W_max_: maximal workload, QF: quadriceps force, HF: handgrip force, CRQ: chronic respiratory disease questionnaire, SGRQ: St George’s respiratory questionnaire, MPA: moderate intense physical activity (daily time spent in activities with an intensity of at least 3 METs).

**Table S3.** Effects of PR and at 1-year on maximal exercise capacity and muscle force.

|  | *Control* | *Rehabilitation* | *Rehabilitation*  *effect (95%IC)* | *p- Value* |
| --- | --- | --- | --- | --- |
| W_max_ (W) |  |  |  |  |
| Baseline | 85 (28) | 86 (28) |  | 0,93 |
| 3 months | 81 (28) | 93 (28) | 12 (4, 19) | **0,003** |
| 6 months | 78 (28) | 96 (28) | 16 (7, 26) | **<0,001** |
| 1 year | 75 (28) | 99 (28) | 23 (12, 33) | **<0,001** |
| QF (Nm) |  |  |  |  |
| Baseline | 104,4 (36,7) | 127,6 (36,7) |  | 0,05 |
| 3 months | 104,2 (36,7) | 145,4 (36,7) | 18 (6,6, 29,5) | **0,002** |
| 6 months | 108,3 (36,7) | 149,3 (36,7) | 17,9 (3,3, 32,4) | **0,02** |
| 1 year | 110,6 (36,7) | 148,6 (36,7) | 14,9 (-1,7, 31,4) | 0,08 |
| HF (N) |  |  |  |  |
| Baseline | 27,8 (10,1) | 31,6 (10,1) |  | 0,20 |
| 3 months | 28,9 (10,1) | 37,4 (10,1) | 4,6 (1,5, 7,7) | **0,004** |
| 6 months | 28,5 (10,1) | 36,6 (10,1) | 4,4 (1,1, 7,6) | **0,009** |
| 1 year | 30,2 (10,1) | 36,2 (10,1) | 2,3 (-1,3, 5,8) | 0,21 |
| MIP (cmH_2_O) |  |  |  |  |
| Baseline | 82,6 (22,5) | 84,3 (22,5) |  | 0,81 |
| 3 months | 80,7 (22,5) | 96,2 (22,5) | 13,8 (5, 22,5) | **0,002** |
| 6 months | 87,2 (22,5) | 102,1 (22,5) | 13,2 (2,3, 24) | **0,02** |
| 1 year | 86,7 (22,5) | 98,5 (22,5) | 10 (-2,4, 22,3) | 0,11 |
| MIP (%pred) |  |  |  |  |
| Baseline | 93 (17) | 88 (17) |  | 0,54 |
| 3 months | 92 (17) | 103 (17) | 16 (6, 26) | **0,003** |
| 6 months | 99 (17) | 111 (17) | 16 (3, 29) | **0,02** |
| 1 year | 98 (17) | 106 (17) | 13 (-3, 28) | 0,11 |
| MEP (cmH_2_O) |  |  |  |  |
| Baseline | 127,2 (28,7) | 151 (28,7) |  | 0,06 |
| 3 months | 130,7 (28,7) | 180,6 (28,7) | 26,1 (11,9, 40,2) | **<0,001** |
| 6 months | 140,5 (28,7) | 174,7 (28,7) | 10,3 (-8,4, 29,1) | 0,28 |
| 1 year | 138,4 (28,7) | 174 (28,7) | 11,8 (-10,5, 34,1) | 0,30 |
| MEP (%pred) |  |  |  |  |
| Baseline | 86 (16) | 95 (16) |  | 0,15 |
| 3 months | 88 (16) | 115 (16) | 17 (8, 27) | **<0,001** |
| 6 months | 95 (16) | 110 (16) | 6 (-6 ,18) | 0,35 |
| 1 year | 95 (16) | 110 (16) | 5,8 (-8, 19) | 0,40 |

Data are expressed as mean (SD) from the mixed model. The rehabilitation effect at each time point is the difference (and its 95% confidence interval) between changes from baseline in the rehabilitation group and in the control group. W_max_: maximal workload. QF: quadriceps force, HF: handgrip force, MIP: maximal inspiratory pressure, MEP: maximal expiratory pressure. The p-Value on the baseline line assesses differences in baseline values; the other ones assess the effect of rehabilitation at each time point.

**Table S4.** Effects of PR and at 1-year on quality of life.

|  | *Control* | *Rehabilitation* | *Rehabilitation*  *effect (95%IC)* | *p- Value* |
| --- | --- | --- | --- | --- |
| CRQ total |  |  |  |  |
| Baseline | 86 (16) | 88 (16) |  | 0,88 |
| 3 months | 83 (16) | 97 (16) | 15 (6, 23) | **<0,001** |
| 6 months | 81 (16) | 103 (16) | 22 (14, 31) | **<0,001** |
| 1 year | 79 (16) | 96 (16) | 18 (9, 27) | **<0,001** |
| CRQ dyspnoea |  |  |  |  |
| Baseline | 18 (0) | 18 (0) |  | 0,67 |
| 3 months | 17 (0) | 23 (0) | 7 (5, 9) | **<0,001** |
| 6 months | 17 (0) | 24 (0) | 8 (5, 11) | **<0,001** |
| 1 year | 17 (0) | 23 (0) | 7 (3, 10) | **<0,001** |
| CRQ fatigue |  |  |  |  |
| Baseline | 16 (2) | 17 (2) |  | 0,53 |
| 3 months | 16 (2) | 19 (2) | 1 (-1, 3) | 0,22 |
| 6 months | 16 (2) | 20 (2) | 3 (0,1, 5) | **0,04** |
| 1 year | 16 (2) | 17 (2) | 0,8 (-2, 4) | 0,60 |
| CRQ emotion |  |  |  |  |
| Baseline | 31 (3) | 30 (3) |  | 0,64 |
| 3 months | 30 (3) | 33 (3) | 4 (1, 7) | **0,004** |
| 6 months | 29 (3) | 35 (3) | 7 (3, 11) | **<0,001** |
| 1 year | 28 (3) | 35 (3) | 7,5 (3, 12) | **0,001** |
| CRQ mastery |  |  |  |  |
| Baseline | 20 (4) | 20 (4) |  | 0,71 |
| 3 months | 19 (4) | 22 (4) | 3 (1, 4) | **0,002** |
| 6 months | 19 (4) | 24 (4) | 4 (2, 6) | **<0,001** |
| 1 year | 18 (4) | 22 (4) | 3 (0,3, 5) | **0,03** |
| SGRQ symptoms |  |  |  |  |
| Baseline | 44 (17) | 52 (17) |  | 0,19 |
| 3 months | 51 (17) | 49 (17) | -10 (-20, 0,5) | 0,06 |
| 6 months | 52 (17) | 45 (17) | -15 (-26, -4) | **0,01** |
| 1 year | 45 (17) | 40 (17) | -13 (-25, -2) | **0,03** |
| SGRQ activity |  |  |  |  |
| Baseline | 54 (16) | 56 (16) |  | 0,68 |
| 3 months | 56 (16) | 47 (16) | -11 (-21, -1) | **0,03** |
| 6 months | 56 (16) | 44 (16) | -15 (-25, -4) | **0,007** |
| 1 year | 58 (16) | 46 (16) | -14 (-25, -3) | **0,01** |
| SGRQ impact |  |  |  |  |
| Baseline | 33 (16) | 32 (16) |  | 0,90 |
| 3 months | 36 (16) | 32 (16) | -3 (-9, 3) | 0,37 |
| 6 months | 39 (16) | 28 (16) | -10 (-17, -3) | **0,003** |
| 1 year | 37 (16) | 28 (16) | -9 (-16, -2) | **0,01** |
| MRC dyspnoea |  |  |  |  |
| Baseline | 2,6 (0,1) | 3,0 (0,1) |  | 0,11 |
| 3 months | 2,3 (0,1) | 3,0 (0,1) | 0,3 (0.0, 0.7) | 0,06 |
| 6 months | 2,4 (0,1) | 3,1 (0,1) | 0,3 (-0.1, 0.8) | 0,14 |
| 1 year | 2,4 (0,1) | 2,9 (0,1) | 0,1 (-0.5, 0.6) | 0,85 |

Data are expressed as mean (SD) from the mixed model. The rehabilitation effect at each time point is the difference (and its 95% confidence interval) between changes from baseline in the rehabilitation group and in the control group. CRQ: chronic respiratory disease questionnaire, SGRQ: St George’s respiratory questionnaire. The p-Value on the baseline line assesses differences in baseline values, the other ones assess the effect of rehabilitation at each time point.

**Table S5.** Effects of PR and at 1-year on lung function.

|  | *Control* | *Rehabilitation* | *Rehabilitation*  *effect (95%IC)* | *p- Value* |
| --- | --- | --- | --- | --- |
| DL_CO_ (mmol,min^1^,kgPa^-1^) |  |  |  |  |
| Baseline | 3,38 (1,25) | 3,97 (1,25) |  | 0,11 |
| 3 months | 3,19 (1,25) | 3,9 (1,25) | 0,13 (-0,17, 0,43) | 0,39 |
| 6 months | 3,1 (1,25) | 3,75 (1,25) | 0,07 (-0,3, 0,45) | 0,71 |
| 1 year | 3,04 (1,25) | 3,86 (1,25) | 0,23 (-0,18, 0,65) | 0,27 |
| VC (l) |  |  |  |  |
| Baseline | 2,64 (0,91) | 2,86 (0,91) |  | 0,36 |
| 3 months | 2,64 (0,91) | 2,93 (0,91) | 0,07 (-0,08, 0,21) | 0,35 |
| 6 months | 2,55 (0,91) | 2,86 (0,91) | 0,09 (-0,06, 0,24) | 0,25 |
| 1 year | 2,57 (0,91) | 2,9 (0,91) | 0,11 (-0,05, 0,27) | 0,18 |
| FVC (l) |  |  |  |  |
| Baseline | 2,58 (0,92) | 2,77 (0,92) |  | 0,43 |
| 3 months | 2,58 (0,92) | 2,9 (0,92) | 0,13 (-0,01, 0,27) | 0,06 |
| 6 months | 2,5 (0,92) | 2,86 (0,92) | 0,18 (0,03, 0,32) | **0,02** |
| 12 months | 2,52 (0,92) | 2,87 (0,92) | 0,16 (0,01, 0,32) | **0,04** |
| FVC (%pred) |  |  |  |  |
| Baseline | 79 (22) | 77 (22) |  | 0,78 |
| 3 months | 79 (22) | 81 (22) | 4 (-0,3, 8) | 0,07 |
| 6 months | 77 (22) | 80 (22) | 4 (0,3, 9) | **0,04** |
| 12 months | 77 (22) | 81 (22) | 5 (1, 10) | **0,02** |
| TLC (l) |  |  |  |  |
| Baseline | 3,98 (1,16) | 4,31 (1,16) |  | 0,30 |
| 3 months | 4,05 (1,16) | 4,34 (1,16) | -0,04 (-0,24, 0,16) | 0,70 |
| 6 months | 3,97 (1,16) | 4,42 (1,16) | 0,12 (-0,09, 0,33) | 0,26 |
| 1 year | 3,99 (1,16) | 4,41 (1,16) | 0,1 (-0,13, 0,32) | 0,41 |
| TLC (%pred) |  |  |  |  |
| Baseline | 69 (16) | 69 (16) |  | 0,87 |
| 3 months | 70 (16) | 70 (16) | -1 (-4, 3) | 0,71 |
| 6 months | 69 (16) | 71 (16) | 2 (-1, 6) | 0,25 |
| 1 year | 69 (16) | 71 (16) | 1,8 (-2, 6) | 0,37 |
| PaO_2_ (mmHg) |  |  |  |  |
| Baseline | 76,9 (8,8) | 75,7 (8,8) |  | 0,72 |
| 6 months | 76,2 (8,8) | 75.1 (8,8) | 0,1 (-7, 7,3) | 0,97 |
| 1 year | 72,8 (8,8) | 77.2 (8,8) | 5,6 (-2,5, 13,8) | 0,17 |

Data are expressed as mean (SD) from the mixed model. The rehabilitation effect at each time point is the difference (and its 95% confidence interval) between changes from baseline in the rehabilitation group and in the control group. FVC: forced vital capacity, VC: slow vital capacity, FEV_1_: forced expiratory volume in 1 second, DL_CO_: diffusion capacity for carbon monoxide, TLC: total lung capacity, PaO_2_: partial pressure of oxygen at breathing room air. The p-Value on the baseline line assesses differences in baseline values, the other ones assess the effect of rehabilitation at each time point.

**Table S6.** Effects of PR and at 1-year with penalizing treatment of missing data.

|  | *Control* | *Rehabilitation* | *Rehabilitation*  *effect (95%IC)* | *p- Value* |
| --- | --- | --- | --- | --- |
| 6MWD (m) |  |  |  |  |
| Baseline | 491 (103) | 462 (103) |  | 0,35 |
| 3 months | 477 (58) | 489 (58) | 41 (19, 63) | **<0,001** |
| 6 months | 473 (58) | 492 (58) | 48 (20, 75) | **<0,001** |
| 1 year | 473 (58) | 480 (58) | 36 (5, 66) | **0,02** |
| W_max_  (W) |  |  |  |  |
| Baseline | 85 (29) | 86 (29) |  | 0,93 |
| 3 months | 82 (29) | 90 (29) | 8 (1, 14) | **0,02** |
| 6 months | 80 (29) | 90 (29) | 9 (2, 17) | **0,01** |
| 1 year | 80 (29) | 88 (29) | 8 (0, 16) | **0,04** |
| QF (%pred) |  |  |  |  |
| Baseline | 81 (28) | 78 (28) |  | 0,69 |
| 3 months | 81 (28) | 86 (28) | 9 (3, 15) | **0,01** |
| 6 months | 82 (28) | 87 (28) | 8 (2, 14) | **0,01** |
| 1 year | 86 (28) | 92 (28) | 9 (3, 15) | **0,01** |
| CRQ total |  |  |  |  |
| Baseline | 87 (17) | 86 (17) |  | 0,88 |
| 3 months | 83 (17) | 94 (17) | 12 (4, 19) | **0,01** |
| 6 months | 82 (17) | 97 (17) | 16 (9, 24) | **<0,001** |
| 1 year | 81 (17) | 88 (17) | 9 (1, 16) | **0,02** |

Data are expressed as mean (SD) from the mixed model considering a more penalizing hypothesis: in the intervention group, missing data were assumed to follow the general trend of the control group whereas, in the control group, missing data were assumed to be equal to the baseline values. The rehabilitation effect at each time point is the difference (and its 95% confidence interval) between changes from baseline in the rehabilitation group and in the control group. 6MWD: six-minute walking distance, W_max_: maximal workload, QF: quadriceps force, CRQ: chronic respiratory disease questionnaire. The p-Value on the baseline line assesses differences in baseline values, the other ones assess the effect of rehabilitation at each time point.

**References**

1. Wanger J, Clausen JL, Coates A, Pedersen OF, Brusasco V, Burgos F, Casaburi R, Crapo R, Enright P, van der Grinten CP, et al: **Standardisation of the measurement of lung volumes.** *Eur Respir J* 2005, **26:**511-522.

2. Miller MR, Hankinson J, Brusasco V, Burgos F, Casaburi R, Coates A, Crapo R, Enright P, van der Grinten CP, Gustafsson P, et al: **Standardisation of spirometry.** *Eur Respir J* 2005, **26:**319-338.

3. Macintyre N, Crapo RO, Viegi G, Johnson DC, van der Grinten CP, Brusasco V, Burgos F, Casaburi R, Coates A, Enright P, et al: **Standardisation of the single-breath determination of carbon monoxide uptake in the lung.** *Eur Respir J* 2005, **26:**720-735.

4. Quanjer PH, Tammeling GJ, Cotes JE, Pedersen OF, Peslin R, Yernault JC: **Lung volumes and forced ventilatory flows. Report Working Party Standardization of Lung Function Tests, European Community for Steel and Coal. Official Statement of the European Respiratory Society.** *Eur Respir J Suppl* 1993, **16:**5-40.

5. Brooks D, Solway S, Gibbons WJ: **ATS statement on six-minute walk test.** *Am J Respir Crit Care Med* 2003, **167:**1287.

6. Troosters T, Gosselink R, Decramer M: **Six minute walking distance in healthy elderly subjects.** *Eur Respir J* 1999, **14:**270-274.

7. Ross RM: **ATS/ACCP statement on cardiopulmonary exercise testing.** *Am J Respir Crit Care Med* 2003, **167:**1451; author reply 1451.

8. Jones NL, Makrides L, Hitchcock C, Chypchar T, McCartney N: **Normal standards for an incremental progressive cycle ergometer test.** *Am Rev Respir Dis* 1985, **131:**700-708.

9. Decramer M, Lacquet LM, Fagard R, Rogiers P: **Corticosteroids contribute to muscle weakness in chronic airflow obstruction.** *Am J Respir Crit Care Med* 1994, **150:**11-16.

10. Mathiowetz V, Kashman N, Volland G, Weber K, Dowe M, Rogers S: **Grip and pinch strength: normative data for adults.** *Arch Phys Med Rehabil* 1985, **66:**69-74.

11. American Thoracic Society/European Respiratory S: **ATS/ERS Statement on respiratory muscle testing.** *Am J Respir Crit Care Med* 2002, **166:**518-624.

12. Rochester DF, Arora NS: **Respiratory muscle failure.** *Med Clin North Am* 1983, **67:**573-597.

13. Chang JA, Curtis JR, Patrick DL, Raghu G: **Assessment of health-related quality of life in patients with interstitial lung disease.** *Chest* 1999, **116:**1175-1182.

14. Peng S, Li Z, Kang J, Hou X: **Cross-sectional and longitudinal construct validity of the Saint George's Respiratory Questionnaire in patients with IPF.** *Respirology* 2008, **13:**871-879.

15. Guyatt GH, Berman LB, Townsend M, Pugsley SO, Chambers LW: **A measure of quality of life for clinical trials in chronic lung disease.** *Thorax* 1987, **42:**773-778.

16. Jones PW, Quirk FH, Baveystock CM: **The St George's Respiratory Questionnaire.** *Respir Med* 1991, **85 Suppl B:**25-31; discussion 33-27.

17. Jones PW, Quirk FH, Baveystock CM, Littlejohns P: **A self-complete measure of health status for chronic airflow limitation. The St. George's Respiratory Questionnaire.** *Am Rev Respir Dis* 1992, **145:**1321-1327.

18. Yorke J, Jones PW, Swigris JJ: **Development and validity testing of an IPF-specific version of the St George's Respiratory Questionnaire.** *Thorax* 2010, **65:**921-926.
